# De novo transcriptome assembly, polymorphic SSR markers development and population genetics analyses for southern corn rust (*Puccinia polysora*)

**DOI:** 10.1038/s41598-021-97556-1

**Published:** 2021-09-09

**Authors:** Qiuyu Sun, Jie Liu, Keyu Zhang, Chong Huang, Leifu Li, Jiayu Dong, Yong Luo, Zhanhong Ma

**Affiliations:** 1 College of Plant Protection, China Agricultural University, Beijing, 100193 People’s Republic of China; 2 National Agro-Tech Extension and Service Center, Beijing, 100125 People’s Republic of China

**Keywords:** Genetics, Population genetics, Microbiology, Fungi

## Abstract

Southern corn rust is a destructive maize disease caused by *Puccinia polysora* Underw that can lead to severe yield losses. However, genomic information and microsatellite markers are currently unavailable for this disease. In this study, we generated a total of 27,295,216 high-quality cDNA sequence reads using Illumina sequencing technology. These reads were assembled into 17,496 unigenes with an average length of 1015 bp. The functional annotation indicated that 8113 (46.37%), 1933 (11.04%) and 5516 (31.52%) unigenes showed significant similarity to known proteins in the NCBI Nr, Nt and Swiss-Prot databases, respectively. In addition, 2921 (16.70%) unigenes were assigned to KEGG database categories; 4218 (24.11%), to KOG database categories; and 6,603 (37.74%), to GO database categories. Furthermore, we identified 8,798 potential SSRs among 6653 unigenes. A total of 9 polymorphic SSR markers were developed to evaluate the genetic diversity and population structure of 96 isolates collected from Guangdong Province in China. Clonal reproduction of *P. polysora* in Guangdong was dominant. The YJ (Yangjiang) population had the highest genotypic diversity and the greatest number of the multilocus genotypes, followed by the HY (Heyuan), HZ (Huizhou) and XY (Xinyi) populations. These results provide valuable information for the molecular genetic analysis of *P. polysora* and related species.

## Introduction

Southern corn rust caused by *Puccinia polysora* is one of most devastating fungal diseases of corn; it was first described and named by Underw in 1897^1^. SCR is distributed in tropical, subtropical and temperate cultivation regions, including Asia^2^, the United States^3,4^, Australia^5^ and Africa^1,6^. An increase in disease occurrence has been reported in recent years in China. The lack of varieties resistant to SCR and long-distance migration of the disease are the main reasons for severe disease epidemics^3,7,8^.

SCR appears to have great potential to damage plants. Symptoms initially appear on leaves and expand through an entire plant, including the leaves, stalks, leaf sheaths and husks, in late-season planting, which can result in leaf necrosis and complete destruction of photosynthetic areas, followed by death of the plant^9–12^. The risk of the pandemics of this disease is a big threat^11^. Although several physiologic races of *P. polysora* have been described^13–17^ and resistance has been reported^3,7^, the majority of commercial hybrids grown in China and the United Stated are rated as susceptible to *P. polysora*^18–20^. In the past, the disease has caused several serious yield losses in various countries, including West Africa (50%^6^), the Philippines (80–84%^21^), Brazil (40%^22^), the United States (30–50%^3^) and China (42–53%^23^).

The life cycle and mating system of *P. polysora* remain unknown. The alternate host of *P. polysora* has never been found. Urediniospores serve as both primary and secondary inoculum sources of mini cycle of the disease^2^. The aecidial and pycnidial stages of *P. polysora* are absent^9^. Teliospores are rarely or not produced^2^. All experiments attempting to induce the germination of teliospores have been unsuccessful^2,9^. The function of teliospores in the life cycle remains mystery, although they could be of significance in assuring extended survival of the pathogen. The highly successful uredo stage and the ease of continuity of this stage may have resulted in the suppression of the sexual phase in the life cycle^9^. Due to the lack of evidence, Cammack^9^ provisionally classified *P. polysora* as a microcyclic and autoecious hemiform.

Although the disease is a main problem of corn production, few nucleotide sequences of *P. polysora* have been deposited in GenBank. The lack of whole-genome sequences means that little molecular information is available for the pathogen, which limits the research on this pathogen. Illumina sequencing of the transcriptomes of model and nonmodel organisms previously confirmed that short reads can be effectively assembled^24–26^. For nonmodel organisms with limited genomic information, transcriptome sequencing, which focuses on the sequencing of functional and protein-coding RNAs, is a cost-effective method^27,28^. Transcriptome data are invaluable in the discovery of gene functions, metabolic pathways and molecular markers^25^.

Polymorphic simple sequence repeats (SSRs) have been proven to be important for assessing genetic diversity and population structure^29,30^, however, no SSR markers for *P. polysora* have been developed and reported to date. Without effective molecular markers, the genetic diversity of *P. polysora* has rarely been reported. The population diversity of *P. polysora* was investigated using restriction fragment length polymorphisms (RFLPs) in Japan^31^ and inter-simple sequence repeats (ISSRs) in Thailand^32^ and China^33^. The traditional and standard methods for developing SSR markers are time-consuming and expensive, whereas transcriptome sequencing provides a high-throughput source for investigating SSRs^34,35^.

The objectives of this study were to (1) sequence the transcriptomes of urediniospores of *P. polysora* using an RNA-seq sequencing platform, Illumina HiSeq2000, (2) assemble and annotate the unigenes of *P. polysora*, (3) develop polymorphic SSR markers, and (4) genotype and characterize the population genetics of *P. polysora* isolates collected from the cities of Xinyi, Huizhou, Yangjiang and Heyuan in Guangdong Province. The transcriptome sequences provide a valuable genomic resource for the molecular study of *P. polysora*, and the findings provide the first perspective into the *P. polysora* population structure in Guangdong Province.

## Results

### Sequence assembly

**Figure 1:**
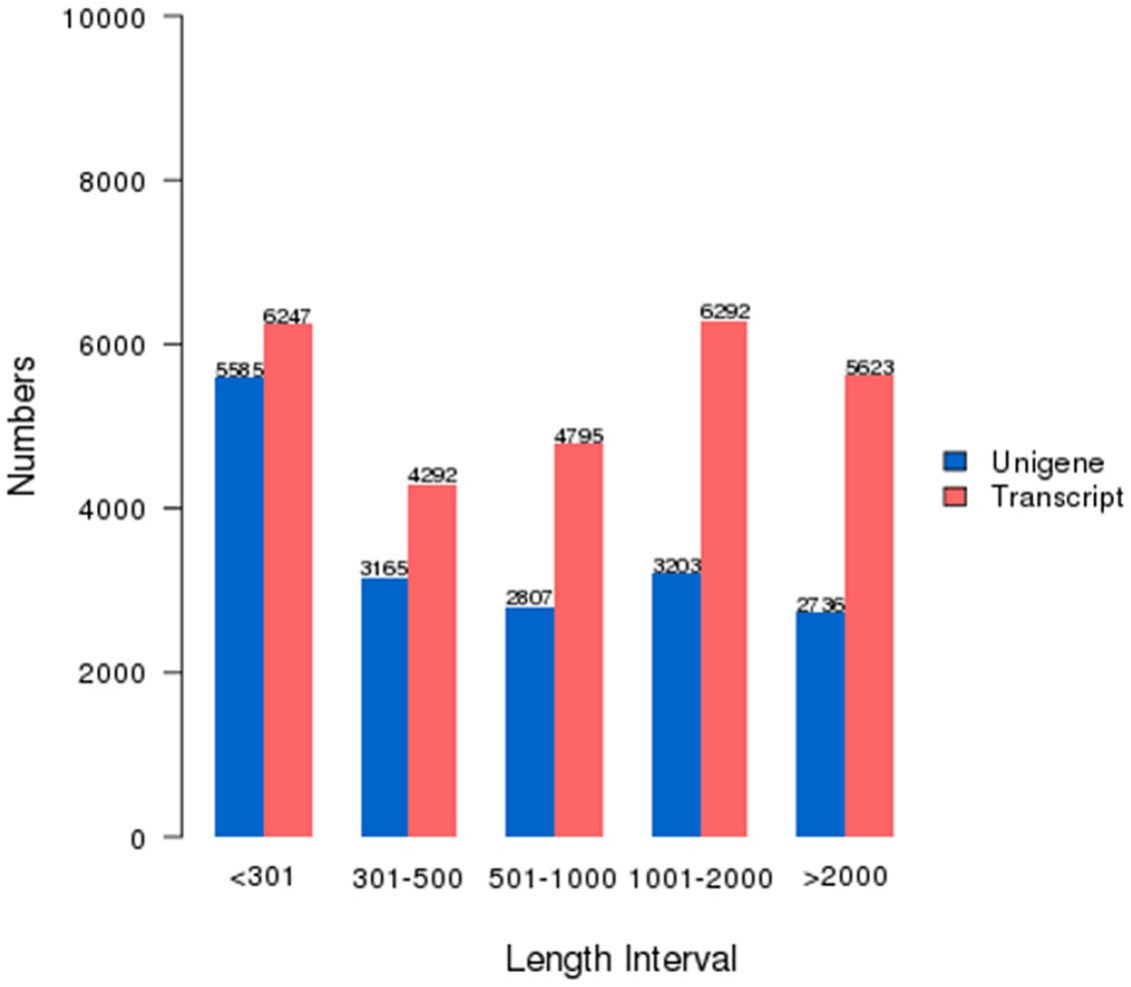
The transcript and unigene length distribution *of P. polysora*.

We sequenced the transcriptomes of urediniospores of *P. polysora.* A total of 28,252,282 raw reads (4.09 Gbp) were obtained. The percentages of Q20 and Q30 were 96.62% and 91.76%, respectively. The GC content was approximately 49.49%. After quality control, the remaining high-quality reads were assembled into 10,539 transcripts with an average length of 1,229 bp and an N50 of 2,083 bp (Table 1). We obtained 17,496 unigenes varying from 201 bp to 15,021 bp with an average of 1,015 bp and an N50 of 1,922 bp (Table 1). The length distribution of the unigenes showed that 10,539 unigenes were 201–500 bp, 4,795 unigenes were 500–1,000 bp, 6,292 unigenes were 1,000–2,000 bp and 5,623 unigenes were longer than 2,000 bp (Fig. 1).

**Table 1:** Summary of sequence assembly.

	Min length	Mean length	Median length	Max length	N50	N90	Total nucleotides	Total number
Transcripts	201	1229	782	15,021	2083	523	33,498,329	27,249
Unigenes	201	1015	500	15,021	1922	362	17,765,396	17,496

### Functional annotation

The functional annotation of all the unigenes was conducted by searching against published databases. Based on the sequence similarity, a total of 9550 (54.58%) unigenes were annotated. Because of the lack of genome information for *P. polysora*, 7946 unigenes could not be matched to known genes. The overall functional annotation across each database is described in Table 2.

**Table 2:** Summary of functional annotation of unigenes of *P. polysora*.

Annotation database	Number of unigenes	Percentage (%)
Annotated in Nr	8113	46.37
Annotated in Nt	1933	11.04
Annotated in Swiss-Prot	5516	31.52
Annotated in Pfam	6128	35.02
Annotated in GO	6603	37.74
Annotated in KOG	4218	24.1
Annotated in all database	858	4.9
Annotated in at least one database	9550	54.58
Total Unigenes	17,496	100

**Figure 2:**
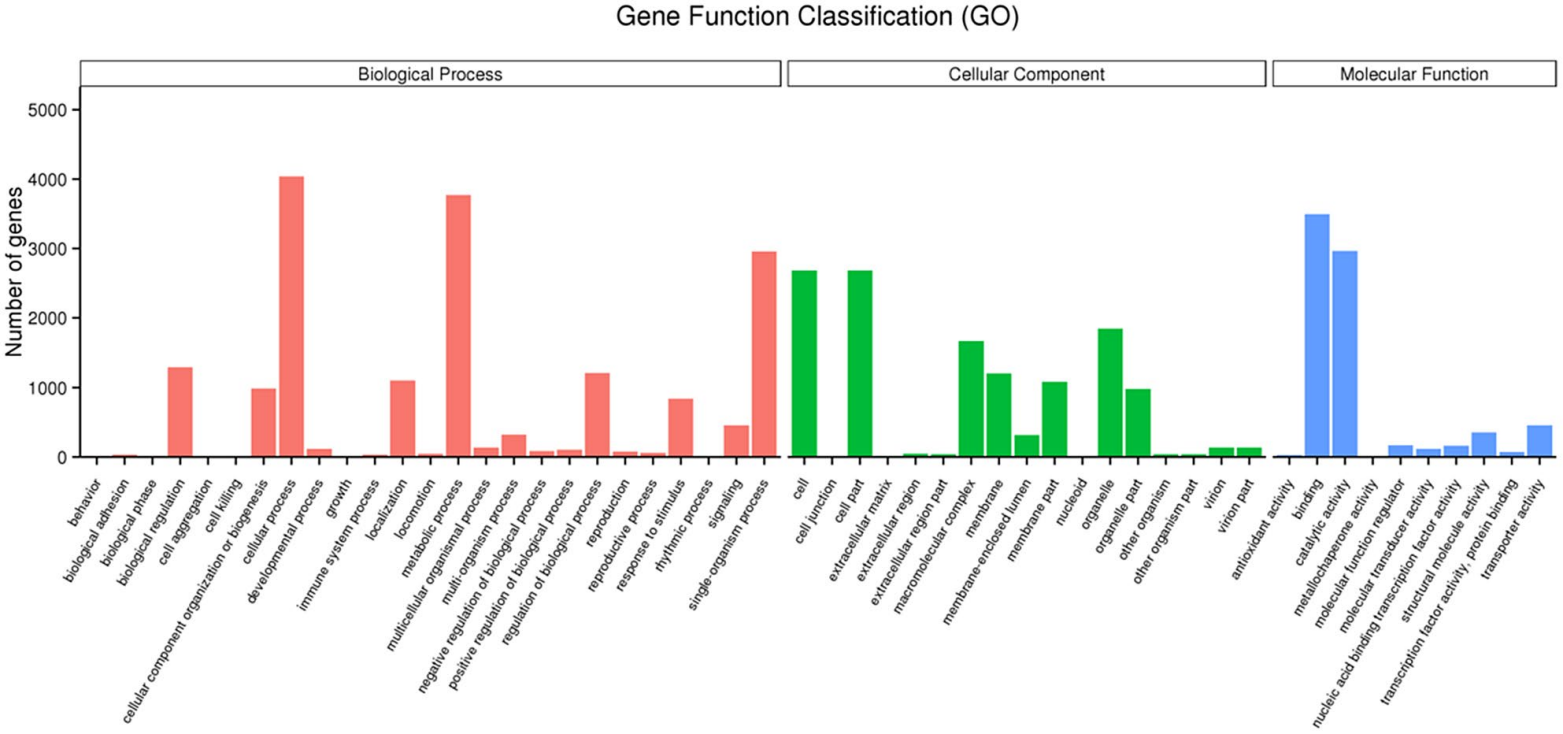
Gene ontology (GO) classification *of P.polysora* unigenes. A total of 6603 unigenes with significant similarity in Nr database categorized into three main GO categories.

There were 6,603 unigenes categorized into three main GO categories: biological process (17,725), cellular component (7,824) and molecular function (12,942) (Fig. 2, Table S1). Within the three categories, ‘cellular process’ (4,040), ‘binding’ (3,495), ‘cell part’ (2,685) and ‘cell’ (2,685) were the most prevalent. A high percentage of genes as classified under the ‘single-organism process’ (2,958), ‘catalytic activity’ (2,964) and ‘metabolic process’ (3,772) terms. The categories ‘metallochaperone activity’ (3), ‘cell aggregation’ (1) and ‘cell killing’ (2) represented the smallest groups.

A total of 4,218 unigenes were clustered into 25 orthologous groups (KOG/COG) (Fig. S1, Table S2). Among these categories, the cluster ‘General function prediction only’ (567) was the largest group, followed by the cluster ‘Translation, ribosomal structure and biogenesis’ (533) and ‘Posttranslational modification, protein turnover, chaperones’ (477), while ‘Cell motility’ (3) and ‘Extracellular structures’ (2) represented the smallest groups.

**Figure 3:**
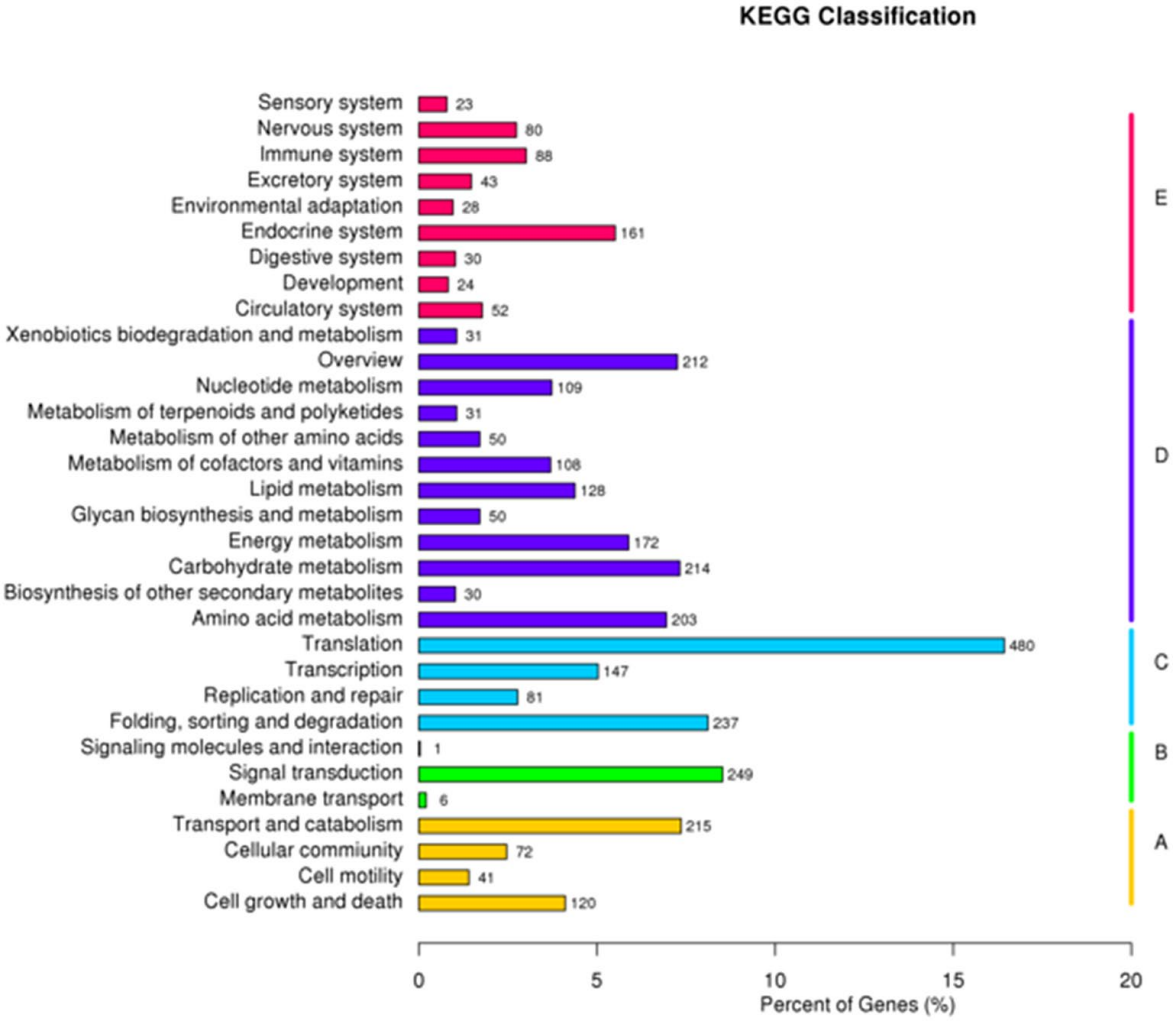
Pathway assignment based on the Kyoto Encyclopedia of Genes and Genomes (KEGG). (**A**) Cellular process. (**B**) Environmental information processing. (**C**) Genetic information processing. (**D**) Metabolism. (**E**) Organismal systems.

To further analyse the transcriptome of *P. polysora*, all the unigenes were analysed in the KEGG pathway database. A total of 2,921 unigenes with significant matches were assigned to 32 KEGG pathways in five main categories (Fig. 3, Table S3). Among these five main categories, ‘Translation’ (480) constituted the largest category, followed by ‘Signal transduction’ (249), ‘Sorting and degradation’ (237) and ‘Amino acid metabolism’ (203).

### SSR marker detection

After a bibliographic research, we have not found any SSR markers for *P. polysora*; therefore, we detected novel SSRs. In this study, all the assembled unigenes were used to mine potential SSRs. We identified a total of 8,798 potential SSRs in 6,653 unigenes (Table S4, Table S5). There were 2,157 unigenes containing more than one SSR. The largest fraction of identified SSR markers consisted of mononucleotides, which accounted for 52.4% (4,609), followed by those with trinucleotide repeats (2,461), dinucleotide repeats (1,351) and tetranucleotide repeats (290) (Table 3). In the mononucleotide repeats, the dominant repeat motif was A/T (3,631), followed by C/G (978). For the dinucleotide repeats, the AG/CT (898) motif was much more common than AC/GT (252), AT/AT (198) and CG/CG (3). For the trinucleotide repeats, the most common motif was ATC/ATG (826), followed by AGG/CCT (375), ACC/GGT (312) and AAG/CTT (278) (Table 3).

**Table 3:** Summary of SSRs identified from the transcriptome of *P. polysora*.

SSR type	Repeats	Total number	Proportion of total SSRs (%)
Mono-nucleotide	Total	4,609	0.52
	A/T	3,631	0.41
	C/G	978	0.11
Di-nucleotide	Total	1,351	0.15
	AC/GT	252	0.03
	AG/CT	898	0.10
	AT/AT	198	0.02
	CG/CG	3	0.00
Tri-nucleotide	Total	2,461	0.27
	AAC/GTT	201	0.02
	AAG/CTT	278	0.03
	AAT/ATT	52	0.01
	ACC/GGT	312	0.04
	ACG/CGT	209	0.02
	ACT/AGT	110	0.01
	AGC/CTG	79	0.01
	AGG/CCT	375	0.04
	ATC/ATG	826	0.09
	CCG/CGG	19	0.00
Tetra-nucleotide	Total	290	0.03
Pentanucleotide	Total	39	0.00
Hexanucleotide	Total	48	0.01

### Identification of novel polymorphic SSR markers

Based on their flanking sequences, a total of 10,440 primer pairs for 3,480 out of 8,798 potential SSRs were successfully designed. We selected 400 high-quality SSR primer pairs for amplification and polymorphism detection via PCR with 96 isolates of *P. polysora* sampled in Guangdong Province. Of the primers we designed, 9 primers produced polymorphic fragments with the 96 isolates tested (Table 4). For the polymorphic loci, the number of alleles per locus ranged from 2 to 4, with an average of 3.0 alleles per locus. Nei’s gene diversity (*H*_*exp*_), which estimates gene diversity, ranged from 0.021 to 0.502^36^. Simpson’s index λ (the genotypic diversity index) ranged from 0.021 to 0.499, and evenness ranged from 0.353 to 0.998 (Table 4)^37^. Locus Ppoly8 had the highest Simpson diversity (0.499) and the most evenly distributed alleles (0.998). A genotype accumulation curve showed that these 9 loci are informative for population genetic analysis (Fig. S2).

**Table 4:** Polymorphic microsatellite primers of *P. polysora*. *Locus* represent each SSR. *Motif* is the repeat motif of each SSR. *F* is the forward primer and *R* is the reverse primer of SSR. Forward primers are labeled with fluorescent tags (FAM, HEX or TAMRA). *Nb* (*allele sizes*) is the number of alleles. *Tm* represents annealing temperature. *λ* and *H*_*exp*_ are diversity indices.

*Locus*	*Motif*		Primer sequence	Nb (allele sizes)	*Tm*	*λ*	*H*_*exp*_	*Evenness*
Ppoly1	(GA)7	F:	***TAMRA***-GAATCTGAGCCGAGGTCGAG	4 (172–174–176–178)	59	0.156	0.157	0.439
		R:	CTCAACTCACCACACCCTCC					
Ppoly2	(ATG)6	F:	***HEX***-TCTTGAGTGTTCTGACGGCC	3 (384–392–395)	58	0.229	0.230	0.517
		R:	GAGTAGCCCCCAAGTCATCG					
Ppoly3	(GGC)5	F:	***HEX***-AGAAGAGGCAAACGACCTGG	3 (166–171–174)	57	0.182	0.183	0.482
		R:	AAGTTCACTTCTGGAGGGCG					
Ppoly4	(GA)6	F:	***FAM*-**AAAGGACCGACGCAAGAGAG	4 (261–265–269–271)	57	0.100	0.101	0.390
		R:	CCGATCGAGTCCAATACCCG					
Ppoly5	(AT)6	F:	***TAMRA***-CCTATCTTCTTCGGTCCGCC	3 (258–260–262)	57	0.129	0.129	0.431
		R:	GAGCAGAGAGATAAGTGGCC					
Ppoly6	(GGA)5	F:	***HEX***-AGCGAGGGTCTTGTCATTGG	2 (169–217)	57	0.437	0.440	0.887
		R:	TCCTCATCATCCTCGTCTTCCT					
Ppoly7	(TC)7	F:	***FAM*-**GTGGTCGTGGTGGTGATGAT	3 (252–254–256)	57	0.071	0.071	0.384
		R:	TAGCCAGTCAACAAGCCTCG					
Ppoly8	(CT)5	F:	***FAM*-**AAGCTACTCCACCACCTCCT	2 (166–168)	57	0.499	0.502	0.998
		R:	TCTCATTGCCCTCGAACGAC					
Ppoly9	(GATC)5	F:	***HEX***-CGGTCGCTAGTTCGGATGAC	3 (209–213–217)	59	0.021	0.021	0.353
		R:	CGAGAAGAGGATGGACGACG					
Mean				3.00		0.203	0.204	0.542

### Population genetic diversity

The population genetic diversity of four populations (HY, HZ, XY and YJ) of *P. polysora* was analysed (Table 5). A total of 32 multilocus genotypes (MLGs) were detected in the 96 isolates based on the 9 novel SSR markers. The number of MLGs observed for each population ranged from 6 to 14. To reduce the influence of sample size, a more appropriate comparison, eMLG, was performed. eMLG is a measure of the number of genotypes that would be expected at the largest, shared sample size based on rarefaction. The YJ population had the highest genotypic diversity and the most eMLGs, followed by HZ, HY and XY. In addition, YJ was also the most even population, followed by XY, HZ and HY. Except for the XY population, the index of association $\overline{r}_{d}$ and the standardized index of association *I*_*A*_ showed that *P. polysora* reproduced clonally.

**Table 5:** Genotypic diversity statistics of four populations in Guangdong Province for *P. polysora*. *N* is the number of isolates in each population. *MLG* is the number of the multilocus genotypes observed. *eMLG* is the number of expected MLG. *SE* is the standard error based on eMLG. The next four columns are diversity indices. *I*_*A*_ and $\overline{r}_{d}$ are the index of association and the standardized index of association, respectively. *P < 0.05.

*Pop*	*N*	*MLG*	*eMLG*	*SE*	*H*	*G*	*λ*	*H*_*exp*_	*Evenness*	*I*_*A*_	$\overline{r}_{d}$
HY	36	14	6.09	1.37	1.72	2.81	0.64	0.21	0.40	2.48*	0.38*
HZ	34	13	6.20	1.34	1.87	4.19	0.76	0.16	0.58	0.56*	0.10*
XY	13	6	6.00	0.00	1.41	2.96	0.66	0.17	0.63	0.06	0.02
YJ	13	10	10.00	0.00	2.20	8.05	0.88	0.32	0.87	1.29*	0.18*
Total	96	32	6.68	1.56	2.28	4.21	0.76	0.20	0.37	1.64*	0.22*

### Population structure

**Figure 4:**
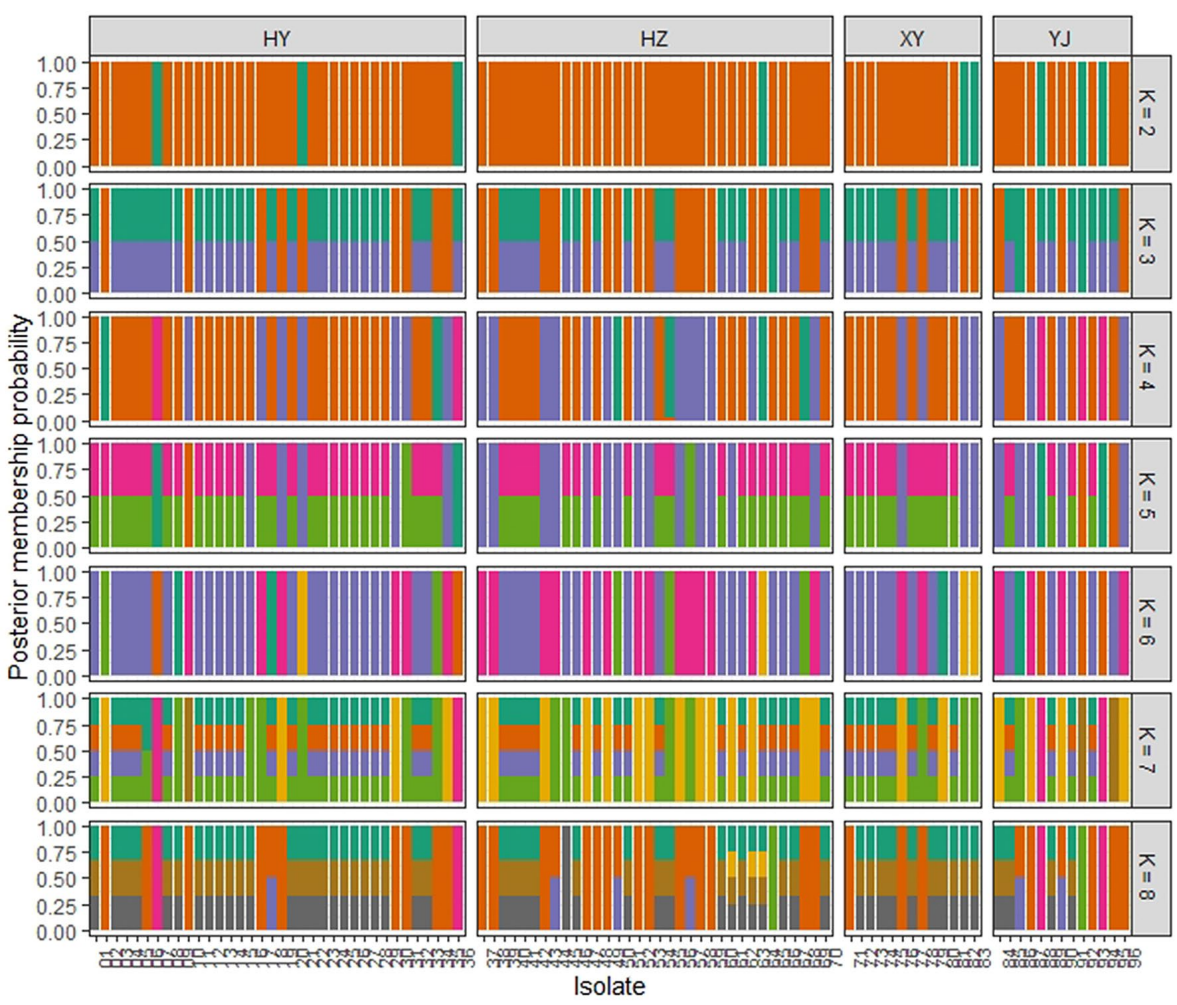
Discriminant analysis of principal components (DAPC) of the 96 *P. polysora* isolates. Posterior membership probabilities of each isolate based on the discriminant functions of DAPC. The group K was set from 2 to 8. Each isolate is represented by a vertical bar.

Discriminant analysis of principal components (DAPC) was performed to analyse the genetic structure of 96 *P. polysora* isolates. The optimal number of PCs (30) was assessed using the *find.clusters* function. The cluster membership probabilities of each isolate based on the discriminant functions of DAPC were conducted with cluster numbers (K) from 2 to 8 (Fig. 4). With the increase in cluster number, population subdivision was gradually generated among the populations. All the populations in Guangdong Province exhibited admixture with other populations, and YJ showed the most admixture.

**Figure 5:**
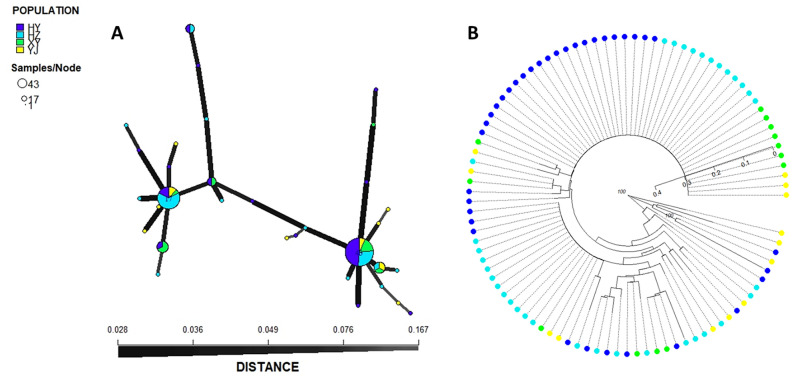
Minimum spanning network (MSN) and neighbour-joining (NJ) tree of 96 *P. polysora* isolates. Samples are colored according to the cities they were collected from. (**A**) MSN is constructed using the genetic distance of Bruvo. All the mutilocus genotypes are shown as a circle. The circle scale shows the number of isolates sharing the same mutilocus genotype. The ratio of colors in each circle is proportional to the ratio of isolates collected in different cities with the same mutilocus genotype. (**B**) Neighbour-joining (NJ) tree of 96 *P. polysora* isolates. Only values greater than 75 are shown based on 999 bootstraps performed.

A neighbour-joining (NJ) phylogenetic tree and minimum spanning network (MSN) were conducted to further assess the population structure of *P. polysora*. The structure of *P. polysora* was dominated by the presence of two clonal linages (Fig. 5B). There were 44 and 17 isolates from all the populations clustered into these two clonal linages, respectively. Most isolates with high genetic distance from the YJ population were clustered into groups with cryptic diversity. The MSN results showed that two dominant MLGs were observed in all the populations (Fig. 5A). Populations HY and HZ contributed substantially to the major groups of MLGs, and HY had more unique MLGs than the other populations. The MSN results were consistent with the NJ tree analysis.

## Discussion

Recently, southern corn rust has become research focus because of its wide distribution, great damage and high incidence in China. Although southern corn rust is a devastating disease worldwide, the complete genome sequence and transcriptomic data for *P. polysora* remain unknown. Transcriptome analysis is an attractive alternative to examine the properties of a transcriptome as a proxy for the whole genome^38^. Transcriptome sequencing is a powerful and cost-effective method for generating large-scale transcriptomes and is used to annotate novel genes for model and nonmodel organisms and to develop molecular markers^24,25,26^. To our knowledge, the present study is the first attempt to perform de novo assembly of the transcriptome and to develop SSR markers of *P. polysora*.

In this study, we obtained a total of 27,295,216 high-quality reads with a Q20 of 96.62% using Illumina paired-end sequencing. A total of 17,496 unigenes were assembled, which is similar to the gene numbers predicted for other rust fungi, such as *Melampsora larici-populina* (16,399^39^), *M. lini* (16,271^40^) and *P. striiformis* (20–25,000^41,42^). The average length of the unigenes with an N50 of 1,922 bp was 1,015 bp. The GC content was 49.49%. These results indicated that the transcripts of *P. polysora* were of high quality and provided a large amount of valuable transcriptome information for gene annotation, novel gene discovery, and the investigation of molecular evolutionary mechanisms in *P. polysora*^24,25,26^.

To annotate the biological function of unigenes at the transcriptome level, sequence similarity searching was conducted against the Nr, Nt, Swiss-Prot, GO, Pfam, KOG/COG and KEGG databases. More than half of the unigenes (9,550; 54.58%) were matched with known proteins in public databases. Due to the absence of a reference genome for *P. polysora*, almost half of the unigenes might represent novel genes whose function is unknown. In the Nr database, most unigenes (77.00%) were annotated to *Puccinia graminis* f. sp*. tritici,* which causes stem rust of wheat. This result confirmed that *P. polysora* is closely related to *P. graminis* which has been completely sequenced^43^. In addition, we categorized 6,603, 4,218 and 2,921 unigenes into GO categories, orthologous groups (KOG/COG) and KEGG pathways, respectively, indicating that one-third of the unigenes could be well annotated with potential functions. The annotated unigenes might be useful in the investigation of gene function and valuable for further research in the future. These results again demonstrated that genes can be investigated by transcriptome analysis for nonmodel plant species.

In the present research, a total of 8,798 potential SSRs were identified in 6,653 unigenes. We selected 400 high-quality SSR primer pairs for amplification and polymorphism detection via PCR with 96 isolates of *P. polysora*. Only 9 primer pairs with the expected size were polymorphic among the 96 isolates of *P. polysora*. The primer pairs resulting in PCR products larger or smaller than expected may be due to the presence of introns, insertions or repeats, a lack of specificity, or assembly errors^44^. The low percentage of polymorphic loci may be due to the close geographic origin of isolates or the clonal reproduction of the pathogen^45^.

Previous genetic diversity studies of *P. polysora* populations fully support our results^32,33^. Using ISSR markers, Unartngam et al.^32^ found that *P. polysora* isolates from different provinces in Thailand have similar genetic characteristics and are present in the same groups, likely resulting from spore migration. The same result was found in Chinese populations using ISSR markers^33^. The urediniospores of *P. polysora* can be dispersed by air currents up to altitudes greater than 15,000 ft high and spread long distances by wind^1^. The present study again confirmed that the isolates from different localities presented similar genetic characteristics, which might be due to spore migration.

All the populations in Guangdong Province exhibited admixture with other populations and were clustered into two clonal groups, while the YJ population showed the highest genotypic diversity and the most eMLGs. More isolates with unique MLGs were found in the YJ population. The pathogen can survive year-round in the most south city Yangjiang belonging to the tropical zone, which might increase the possibility of new genotypes arising.

Our results confirm the previous study showing that *P. polysora* is a microcyclic and autoecious hemiform pathogen^9,32^. The index of association $\overline{r}_{d}$ and the standardized index of association *I*_*A*_ rejected the null hypothesis of linkage equilibrium in three out of four populations, suggesting high rates of selfing and clonal reproduction. Population XY was the exception and was characterized by only 6 MLGs, of which 5 MLGs were shared with other populations. Eight out of 13 isolates clustered into the clonal groups, and 5 isolates showed slight genetic differentiation. The differences remaining in population XY could lead to not reject the null hypothesis of linkage equilibrium, despite the pathogen truly reproducing clonally. Failure to reject the null hypothesis of linkage equilibrium does not mean the confirmation of sexual reproduction. More isolates in population XY are needed for further confirmation. The majority of isolates were clustered into two dominant clonal groups, which could be additional support for clonality and selfing.

The observed cryptic diversity appeared to be sampling bias. Members of the diverse groups were from HY, HZ and XY; however, most isolates of these populations were clustered into the dominant clonal groups. Furthermore, the index of association $\overline{r}_{d}$ and the standardized index of association *I*_*A*_ showed that the populations HY and HZ are highly clonal or selfing. Therefore, we propose that the observed cryptic diversity is because of sampling bias. Isolates of the cryptically diversity groups likely belong to their own clonal group or groups, that were not sampled deeply enough to obtain their membership.

This work provides novel insights into the transcriptome, annotated unigenes, polymorphic SSRs and population genetics of *P. polysora.* More work is needed to investigate the gene function and population structure of *P. polysora.*

## Methods

### Pathogen materials

There is no research report on the physiological races and pathogenic types of *P. polysora* in China. To investigate the population genetics of *P. polysora* in Guangdong Province, we collected diseased leaves from the cities of Xinyi, Huizhou, Yangjiang and Heyuan in Guangdong Province. In each city, two to ten sampling sites with distances of at least 500 m apart from each other were identified, and 10 diseased leaves were randomly collected in each sampling site (Table 6). The plant materials used in this study were obtained from the wild and permission was obtained to collect samples. The collection of plant materials also complied with institutional, national, or international guidelines and legislation. The voucher specimens were preserved in the public herbarium of the Key Lab of Pest Monitoring and Green Management, Ministry of Agriculture and Rural Affairs of China.

**Table 6:** Summary information of the voucher specimens for *Puccinia polysora* populations. P represents the population. Isolates collected from the same city were considered to be one population.

P	Voucher ID	City	Town	Longitude	Latitude	Number of isolates	Sampling time	Identifier
XY	GD_XYCD	Xinyi	Chidong	110.9250	22.4603	3	Jun 2017	Shouren Lai
XY	GD_XYDZ	Xinyi	Dongzhen	110.9536	22.3601	4	Jun 2017	Shouren Lai
XY	GD_XYDB	Xinyi	Dingbao	111.0179	22.3132	6	Jun 2017	Shouren Lai
HZ	GD_HZXK	Huizhou	Gongzhuang	114.3822	23.5480	6	May 2017	Yishan Huang
HZ	GD_HZZG	Huizhou	Gongzhuang	114.4045	23.5343	2	May 2017	Yishan Huang
HZ	GD_HZPTS	Huizhou	Gongzhuang	114.3910	23.5357	1	May 2017	Yishan Huang
HZ	GD_HZSYW	Huizhou	Gongzhuang	114.3747	23.5270	3	May 2017	Yishan Huang
HZ	GD_HZNX	Huizhou	Gongzhuang	114.6066	23.0688	3	May 2017	Yishan Huang
HZ	GD_HZYG	Huizhou	Pingtan	114.3997	23.5271	6	May 2017	Yongmei Zhang
HZ	GD_HZDL	Huizhou	Yonghu	114.4838	22.9500	2	Jun 2017	Yongmei Zhang
HZ	GD_HZWNP	Huizhou	Yonghu	114.5437	22.9716	5	Jun 2017	Yongmei Zhang
HZ	GD_HZQF	Huizhou	Yonghu	114.4838	22.9500	2	Jun 2017	Yongmei Zhang
HZ	GD_HZQF	Huizhou	Yonghu	114.4838	22.9500	4	Jun 2017	Yongmei Zhang
HY	GD_HYQF	Heyuan	Yuanshan	114.5120	24.4199	5	Jul 2017	Chungen Xie
HY	GD_HYZC	Heyuan	Shangping	114.5984	24.4958	4	Aug 2017	Chungen Xie
HY	GD_HYGQ	Heyuan	Youxi	114.3078	24.5102	11	Jul 2017	Chungen Xie
HY	GD_HYGZ	Heyuan	Neiguan	114.3921	24.4602	3	Jul 2017	Chungen Xie
HY	GD_HYSS	Heyuan	Beitou	114.5716	24.3809	2	Jul 2017	Chungen Xie
HY	GD_HYQS	Heyuan	Shangping	110.2633	22.3612	11	Jul 2017	Chungen Xie
YJ	GD_YJSS	Yangjiang	Hexi	111.7382	22.1657	1	Jun 2017	Xibo Mo
YJ	GD_YJKD	Yangjiang	Hexi	111.7513	22.1983	12	Jun 2017	Xibo Mo

To obtain enough pure urediniospores for RNA extraction, reproduction of *P. polysora* was conducted in a greenhouse. *P. polysora* is an obligate parasite. Three-leaf maize seedlings of susceptible ‘Zhengdan 958’ grown in a pot were rubbed to remove wax and sprayed with water mist. The urediniospores collected from diseased leaves were inoculated on the leaf surface. The inoculated plants were moisturized at 25 to 30 °C for 24 h in a sealed box to promote infection. The inoculated plants were transferred to a greenhouse at 25 to 30 °C. After 10 to 15 days, urediniospores were harvested and used for RNA extraction.

Populations were grouped based on cities and assigned as follows: XY (Xinyi), HZ (Huizhou), YJ (Yangjiang) and HY (Heyuan). A single pustule was collected from the sampled leaf to obtain each isolate on the assumption that each pustule was caused by a single urediniospore. To obtain enough urediniospores for DNA extraction, reproduction of isolates was conducted using the same method described above. A total of 98 isolates were obtained (Tables 5, 6).

### RNA extraction

Total RNA was extracted using a TRIzol Kit (Promega, Beijing, China) following the manufacturer’s instructions. Residual DNA was removed using RNase-free DNase I (Takara Biotech Incorporation, Otsu, Japan) for 30 min at 37 °C. The purified RNA quality and quantity were verified using a spectrophotometer (Thermo Scientific, Waltham, MA, USA) and an Agilent 2100 Bioanalyzer (Agilent Technologies, Santa Clara, CA, USA).

### cDNA library construction and Illumina sequencing

Poly(A) mRNA was enriched by oligo (dT) magnetic beads. All mRNA sequences were cut into short fragments by using fragmentation buffer. Taking the short fragments as templates, random hexamer primers were used to synthesize first-strand cDNA. Then, buffer, dNTPs, polymerase I and RNase H were added to synthesize second-strand cDNA. The cDNA fragments were purified using the AMPure XP system (Beckman Coulter, Beverly, MA, USA), resolved with EB buffer for end repair and A tailing, and connected with sequencing adapters. PCR was performed using Phusion High-Fidelity DNA polymerase, universal PCR primers and an index primer. PCR products were purified using the AMPure XP system and assessed on DNA high-sensitivity chips using the Agilent Bioanalyzer 2100 system to construct the final cDNA library. The cDNA library was sequenced by Illumina HiSeq2000 sequencing performed by Novogene Bioinformatics Technology Co., Ltd., Beijing, China.

### Sequence assembly and gene annotation

The high-quality clean reads for assembly were separated from adapters. Reads with more than 50% Q20 bases and those with more than 10% ambiguous “N” bases were removed. De novo transcriptome assembly was conducted using Trinity software with an optimized k-mer length of 25^46^. Unigenes of the transcriptome were annotated using the NCBI nonredundant protein (Nr) database, NCBI nonredundant nucleotide sequence (Nt) database, Kyoto Encyclopedia of Genes and Genomes (KEGG) database, a manually annotated and reviewed protein sequence (Swiss-Prot) database, Cluster of Orthologous Groups of proteins (KOG/COG) database, Gene Ontology (GO) database and protein family (Pfam) database. The statistical enrichment of the unigenes in KEGG pathways was tested by KOBAS software as shown previously^47,48^. The annotation with the Nr, Nt, Swiss-Prot and KOG databases used NCBI Blast version 2.2.28 + with an e-value of 1e^−5^. GO analysis was performed using Blast2GO version 2.5^49^. Pfam protein domain prediction was conducted using HMMER version 3.0^50^.

### Polymorphic SSR validation and assessment

Microsatellites were identified using MISA software^51^. The parameters were adjusted to identify mono-, di-, tri-, tetra-, penta- and hexanucleotide motifs with a minimum of ten, six, five, five, five and five repeats, respectively. Primer 3 version 2.2.3 was used to design the primer pairs with default settings. Forward primers were labelled with fluorescent tags (FAM, HEX or TAMRA) to improve the separation of overlapping markers during multiplexing. Ninety-six isolates sampled from Guangdong Province were used for PCR amplification and polymorphic identification. Amplifications were performed with a thermal cycler (Eppendorf AG) under the following conditions: initial denaturation at 94 °C for 5 min, followed by 35 cycles of 94 °C for 30 s, annealing temperature (Tm) (Table 1) for 30 s, and 72 °C for 1 min, and a final extension at 72 °C for 10 min. Electrophoresis and visualization of alleles were performed on an ABI 3730 DNA analyser (Applied Biosystems, Carlsbad, CA, USA) by Beijing Tsingke Biotech Co., Ltd. Allele sizes were estimated using GeneMarker version 2.2.0 software and compared to a GS500 (35–500 bp) internal standard.

### Population genetics data analysis

We used various R packages to perform population genetic analysis^52^. The R package *poppr*^53^ was used to calculate the number of MLGs and *eMLGs*, genotypic diversity (the Shannon–Wiener index *H*)^54^, Stoddard and Taylor’s index *G*^55^, and Simpson’s index *λ*^37^, evenness *E*_5_^56^, Nei’s unbiased gene diversity *H*_*exp*_^36^, and index of association $\overline{r}_{d}$ and standardized index of association *I*_*A*_^57^. *eMLG* estimates the number of expected MLGs that share the same sample size based on rarefaction. *H* and *G* both measure richness and evenness, increasing as genotypic richness and evenness increase. *G* weights abundant MLGs more heavily than less abundant MLGs. *λ* is an estimation of the probability that two randomly selected genotypes are different. *E*_5_ is a measure of the distribution of genotype abundances. *E*_5_ is equal to one when a population has equally abundant genotypes and closer to zero when it is dominated by a single genotype. *H*_*exp*_ estimates the average heterozygosity. $\overline{r}_{d}$ and *I*_*A*_ are measures of linkage disequilibrium. Significant disequilibrium is expected due to linkage among loci when a population is clonal, and linkage among loci is not expected in sexually reproducing populations.

The package *ape*^58^ was used to draw a neighbour-joining dendrogram with Prevosti’s distance^59^. Minimum spanning networks (MSNs) were constructed with the genetic distance of Bruvo^60^ using the package *poppr*. Discriminant analysis of principal components (DAPC) was plotted using the packages *adegenet*^61^ and *ade4*^62^.

### Data deposit

The transcriptome has been submitted to the National Center for Biotechnology Information (NCBI). BioSample accession: SAMN18713839.

## Supplementary Information

Supplementary Information 1.

Supplementary Information 2.

Supplementary Information 3.

Supplementary Information 4.

Supplementary Information 5.

Supplementary Information 6.
