# Supplementary material for: De novo transcriptome assembly, polymorphic SSR markers development and population genetics analyses for southern corn rust (Puccinia polysora)
Source: Sci Rep. 2021 Sep 9;11:18029. doi: 10.1038/s41598-021-97556-1 (PMC8429556; doi:10.1038/s41598-021-97556-1)
Supplement: Supplementary file 1 — Supplementary Information 1. [file 41598_2021_97556_MOESM1_ESM.docx]

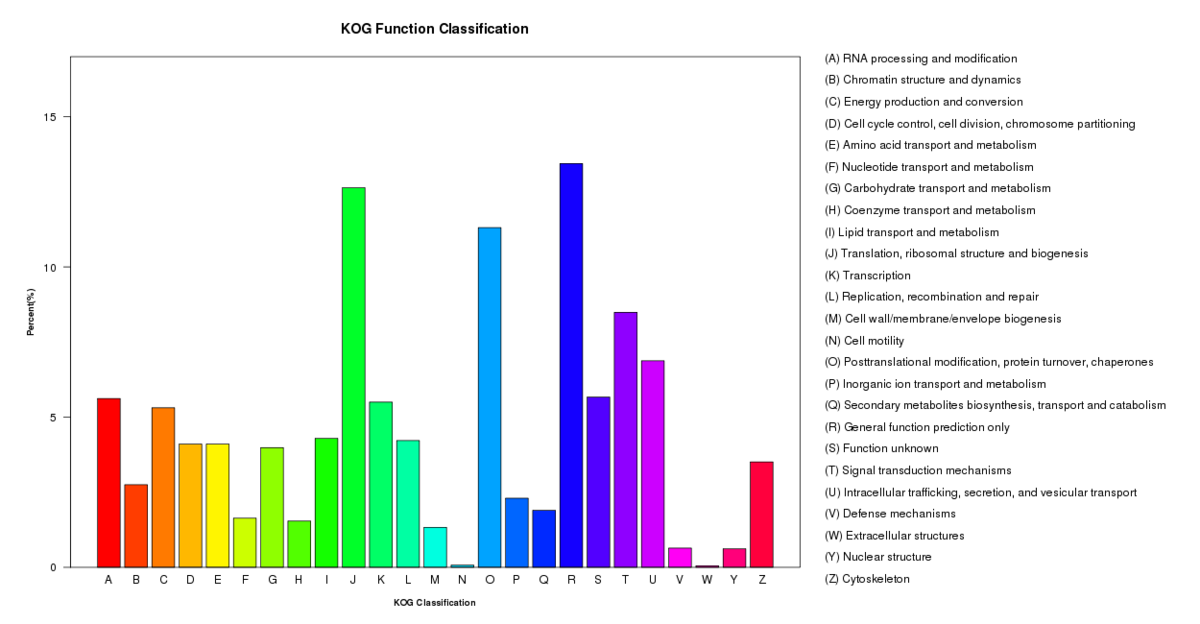


Supplementary Fig. S1. Eukarotic orthologous groups (KOG) classification of *P. polysora* unigenes. A total of 4,218 unigenes were clustered into 25 orthologous groups.


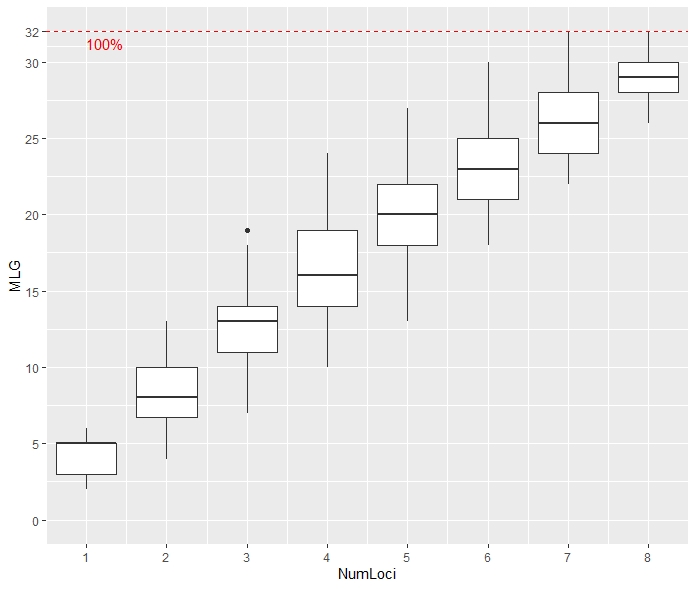


Supplementary Fig. S2. Genotype accumulation curve. For each boxplot, the loci were randomly sampled 1,000 times.
